# Verrucous epidermal nevus

**DOI:** 10.11604/pamj.2023.45.146.40966

**Published:** 2023-08-02

**Authors:** Gerardo Rivera-Silva, María Guadalupe Moreno-Treviño

**Affiliations:** 1 Department of Basic Sciences, School of Medicine, University of Monterrey, Monterrey, Nuevo León, Mexico

**Keywords:** Epidermal nevus, verrucous epidermal nevus, histopathology

## Image in medicine

A 9-year-old male presented with an asymptomatic, confluent, and hyperpigmented skin lesion on the right lateral trunk region presented at birth. No changes in color and consistency are reported, except that the lesion enlarged in proportion to the patient's growth. The mother informed us that the patient was diagnosed with anxiety one year ago. Physical examination revealed a large, hyperpigmented, and confluent patch of overgrown skin with a dimension between 20cm long and 15cm wide associated with painless verrucous plaques located on the right lateral trunk region (A). The laboratory investigations revealed a H1047R mutation in the PIK3CA gene. The anatomopathological study showed hyperkeratosis, acanthosis, and papillomatosis compatible with verrucous epidermal nevi (hamartomas). Anteroposterior (B) and lateral (C) neck radiographs revealed left convexity scoliosis. Based on the clinical, pathologic findings, the lesion was diagnosed as verrucous epidermal nevus (VEN). This malady is benign and congenital and could be associated with abnormalities in neurologic, ophthalmologic, or skeletal systems. When this type of nevi is located on the trunk, is associated with alterations in the bone curvatures of the spine, and/or in the bones of the arms or legs due to mutations in the PIK3CA gene, as in our case. The treatment was a shave excision followed by a phenol peeling medical; although, it is not possible to predict when skin lesions will recur.

**Figure 1:**
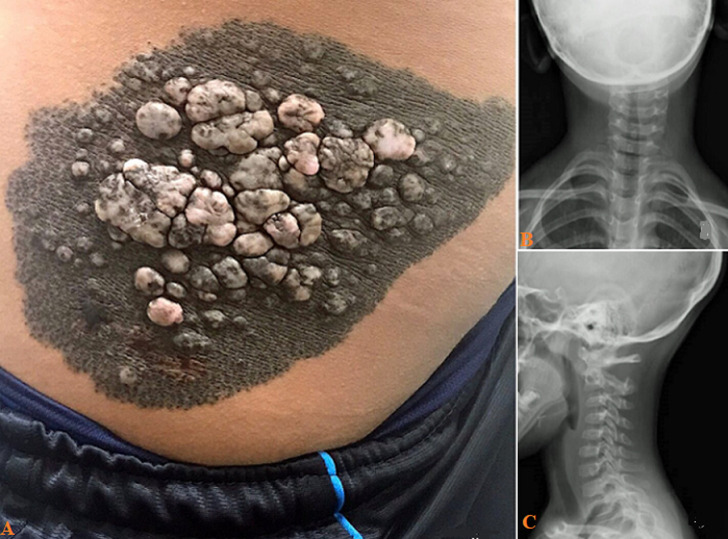
A) general appearance of the nevus showing the hamartomas; B) cervical radiological image of anteroposterior; C) lateral view showing left convexity scoliosis

